# Endosymbiotic bacteria in honey bees: *Arsenophonus* spp. are not transmitted transovarially

**DOI:** 10.1093/femsle/fnw147

**Published:** 2016-06-07

**Authors:** Orlando Yañez, Laurent Gauthier, Panuwan Chantawannakul, Peter Neumann

**Affiliations:** 1 Institute of Bee Health, Vetsuisse Faculty, University of Bern, Schwarzenburgstrasse 161, CH-3003 Bern, Switzerland; 2 Swiss Bee Research Centre, Agroscope, Bern, Switzerland; 3 Bee Protection Laboratory, Department of Biology, Faculty of Science, Chiang Mai University, Chiang Mai 50200, Thailand

**Keywords:** Endosymbionts, *Arsenophonus*, honey bees, *Apis mellifera*, *Apis dorsata*, *Apis florea*

## Abstract

Intracellular endosymbiotic bacteria are common and can play a crucial role for insect pathology. Therefore, such bacteria could be a potential key to our understanding of major losses of Western honey bees (*Apis mellifera*) colonies. However, the transmission and potential effects of endosymbiotic bacteria in *A. mellifera* and other *Apis* spp. are poorly understood. Here, we explore the prevalence and transmission of the genera *Arsenophonus*, *Wolbachia*, *Spiroplasma* and *Rickettsia* in *Apis* spp. Colonies of *A. mellifera* (*N* = 33, with 20 eggs from worker brood cells and 100 adult workers each) as well as mated honey bee queens of *A. cerana*, *A. dorsata* and *A. florea* (*N* = 12 each) were screened using PCR. While *Wolbachia*, *Spiroplasma* and *Rickettsia* were not detected, *Arsenophonus* spp. were found in 24.2% of *A. mellifera* colonies and respective queens as well as in queens of *A. dorsata* (8.3%) and *A. florea* (8.3%), but not in *A. cerana*. The absence of *Arsenophonus* spp. from reproductive organs of *A. mellifera* queens and surface-sterilized eggs does not support transovarial vertical transmission. Instead, horizontal transmission is most likely.

## Abstract (precis)

*Arsenophonus* endosymbiotic bacteria are not transmitted transovarially in honey bees.

## INTRODUCTION

Endosymbiotic bacteria are widespread in arthropods (Hilgenboeker *et al*. 2008; Duron *et al*. 2008). Their interactions with hosts are highly variable ranging from obligate (primary) to facultative (secondary) symbiosis and from parasitic to mutualistic symbiosis (Werren, Skinner and Huger 1986; Perotti *et al*. 2007). The bacteria of the genera *Wolbachia* (Alphaproteobacteria, Rickettsiales), *Spiroplasma* (Mollicutes, Entomoplasmatales)*, Rickettsia* (Alphaproteobacteria, Rickettsiales) and *Arsenophonus* (Gammaproteobacteria, Enterobacteriales) are, in general, facultative endosymbionts and exhibit an extensive host range, including arthropods, nematodes, plants and vertebrates (Bové *et al*. 2003; Perlman, Hunter and Zchori-Fein 2006; Kozek and Rao 2007; Sémétey *et al*. 2007; Bressan *et al*. 2009; Nováková, Hypša and Moran 2009; Wilkes *et al*. 2011).

Transmission of endosymbiotic bacteria to novel hosts is an apparent key element to understand their biological and potential benefits for their hosts. The endosymbionts are usually transmitted vertically. Their spread into the host population can be achieved, in some cases, by manipulating host reproduction, and may cause feminization, cytoplasmic incompatibility and male killing (Werren, Skinner and Huger 1986; Breeuwer and Werren 1990; Werren and O'Neill 1997; Hurst *et al*. 1999; Hurst and Jiggins 2000; Werren, Baldo and Clark 2008; Engelstädter and Hurst 2009). Transmission may be transovarial, in which the bacteria are already present within the eggs (Rollend, Fish and Childs 2013) or transovum, in which the bacteria are present on the eggshells (Prado, Rubinoff and Almeida 2006). Endosymbiotic bacteria can also be transmitted horizontally through contact with infected individuals (Thao and Baumann 2004; Gehrer and Vorburger 2012; Ahmed *et al*. 2013) and from the environment (Bright and Bulgheresi 2010), which is thought to enhance spread to distantly related host species (Russell and Moran 2005; Gehrer and Vorburger 2012).

In honey bees (*Apis* spp.), *Wolbachia* has been detected in workers of *Apis mellifera capensis* and *A. m. scutellata* (Jeyaprakash, Hoy and Allsopp 2003, 2009). Some *Wolbachia* strains have been characterized for *A. m. capensis* (Jeyaprakash, Hoy and Allsopp 2009), but in general virtually nothing is known about effects on host bees. It has been suggested that one of those strains may be responsible for thelytokous parthenogenesis (Hoy *et al*. 2003), but this hypothesis was later rejected (Lattorff, Moritz and Fuchs 2005). Two species of *Spiroplasma*, *Spiroplasma apis* (Mouches *et al*. 1983) and *S. melliferum* (Clark *et al*. 1985), have been characterized, and *S. apis* may be the causal agent of ‘May disease’ (Mouches, Bové and Albisetti 1984). *Rickettsia* in honey bees has been associated with milky hemolymph of infected workers (Wille and Pinter 1961), but later it was shown that the causal agent was filamentous virions (Clark 1978). *Arsenophonus* spp*.* have been detected in the gut microbiota (Babendreier *et al*. 2007; Cornman *et al*. 2012) and in the hemolymph (Gauthier *et al*. 2015) of *A. mellifera* workers and seems to be abundant in the bees’ body surface (Aizenberg-Gershtein, Izhaki and Halpern 2013). Interestingly, *Arsenophonus* spp. appear to be more abundant in colonies displaying clinical symptoms of Colony collapse disorder (CCD; Cornman *et al*. 2012). *Arsenophonus* spp. have also been recently found in *Varroa destructor* (Hubert *et al*. 2015), an ectoparasitic mite, which feeds on the honey bee hemolymph.

Since many endosymbionts may be beneficial for their hosts (Hansen *et al*. 2007; Oliver *et al*. 2010) and may also play a role in honey bee pathology (Evans and Armstrong 2006), it is important to better understand the role of endosymbionts in honey bees in light of *A. mellifera* colony losses (Neumann and Carreck 2010; Aebi and Neumann 2011). Indeed, depending on the strain, *Wolbachia* can protect other hosts against several vectored RNA viruses (Teixeira, Ferreira and Ashburner 2008) and can be regarded as part of host immunity (Zindel, Gottlieb and Aebi 2011). Similarly, *Spiroplasma* rescues host females from the sterilizing effects of nematode parasitism (Jaenike *et al*. 2010). Likewise, other endosymbionts may be beneficial for honey bees. Since even bacterial strains may differ in their effects on hosts, e.g. strains of endosymbiont *Regiella insecticola* differ in their ability to protect pea aphids from parasitoid wasps (Hansen, Vorburger and Moran 2012), it is crucial to also investigate the phylogenetic relationship of the bacteria associated with different species of honey bees. In addition, this may reveal pattern on how these bacteria are interspecifically transmitted. Here, we explore the transmission, prevalence and phylogeny of the endosymbiotic genera *Arsenophonus*, *Wolbachia*, *Spiroplasma* and *Rickettsia* in honey bees *Apis* spp. and focus on transmission of the only detected *Arsenophonus* spp.

## MATERIALS AND METHODS

### Sampling of Asian honey bee queens

Twelve mated queens of *Apis cerana*, *A. dorsata* and *A. florea* each were collected from managed (*A. cerana*) or wild colonies in Chiang Mai and Phatthalung (Thailand), kept in 95% EtOH and stored at –80°C until further analyses.

### Sampling and screening of *Apis mellifera* colonies

Mated *A. mellifera* queens were sampled from colonies that were tested positive for either *Wolbachia*, *Spiroplasma*, *Rickettsia* or *Arsenophonus*. For the screening of local *A. mellifera* colonies (*N* = 33, predominantly *A. m. carnica*), 20 eggs from worker brood cells and 100 adult workers from the middle frames were collected at three apiaries in Bern, Switzerland. All egg samples were homogenized with a sterile plastic pestle in 50μl of Chelex^®^ solution (Bio-Rad, Hercules, CA, USA) for DNA extraction. Samples were incubated at 95°C for 20 min and centrifuged at 12 000 rpm for 2 min. Twenty fold dilutions were used for PCR reactions. DNA was extracted from the pooled worker samples following standard procedures (Evans *et al*. 2013) using the NucleoSpin^®^ Tissue kit (Macherey-Nagel, Dueren, Germany) following the supplier's guidelines. PCR was performed using the high-fidelity Kapa HiFi DNA Polymerase Kit (Kapa Biosystems, Woburn, MA, USA) following the manufacturer's recommendations. Primers and PCR conditions were obtained from previous publications (Table S1, Supporting Informaiton). Parallel amplification of the honey bee *Lys-1* gene (Harpur and Zayed 2013) was used to verify the DNA quality. Negative and positive controls were included in the analyses. PCR products were stained using GelRed for 30 min after electrophoresis in 1.2% agarose gel. Bands were visualized under UV light.

### Queen dissections and screening assays

Laying queens were sampled from *A. mellifera* colonies, which were found positive for any of the tested bacteria (see above). The ovaries and digestive tracts of five *A. mellifera* queens were dissected following standard procedures (Carreck *et al*. 2013). The remains of the queen's bodies were preserved for further analyses. The ovaries, spermathecae, digestive tracts, thoraces and heads from additional three *A. mellifera* queens were also dissected. DNA was extracted from the dissected queen's body parts and the whole bodies of surface sterilized *A. cerana*, *A. dorsata* and *A. florea* queens (Table S2, Supporting Informaiton). Samples were homogenized using a Mixer Mill MM 300 (RETSCH GmbH, Haan, Germany) machine in TN buffer with 3-mm metal beads. DNA extraction and PCR reactions were performed as detailed before. Positive PCR products were Sanger sequenced to ascertain the endosymbiont identity based on 98%–99% BLAST similarity. The *Arsenophonus* spp. sequences derived from ten queens (eight *A. mellifera*, one *A. dorsata* and one *A. florea*) were submitted to the European Nucleotide Archive (ENA) under the accession numbers LN555525-29 and LN890581-85.

### Quantification of *Arsenophonus* spp.

Quantitative real-time PCR (qPCR) was used for the quantification of *Arsenophonus* sp*.* in *A. mellifera* queens. Reactions were performed in triplicate, in a total 12 μl final volume containing 20 ng of template DNA, 6 μl of 2X qPCR Master Mix and 0.2 μM of the forward and reverse primers, using the Kapa SYBR^®^ Fast qPCR kit (Kapa Biosystems, Woburn, MA, USA). Primers were designed from the outer membrane protein assembly factor (*yaeT*) gene (Table S1, Supporting Informaiton). The cycling profile of the real-time qPCR consisted of 30 s incubation at 95°C, 40 cycles of 3 s at 95°C and 30 s at 57°C for annealing, extension and data collection. The melting-curve analysis was performed with the following conditions: 15 s at 95°C, 55°C and 95°C, respectively. Five 10-fold dilutions (10^−2^–10^−6^ ng) of purified amplicons functioned as standards for calibration curve in triplicates (R^2^: 0994; Slope: –3455; Intercept: 36 684; PCR efficiency: 1.947). *A. mellifera* 18S rRNA was used as a reference gene to normalize for extraction efficiency (Ward *et al*. 2007; Table S1, Supporting Informaiton) Software ECO real-time PCR system (Illumina, San Diego, CA, USA) was used to evaluate the performance of the qPCR reactions and to analyze the qPCR quantification.

### Transmission pathway of *Arsenophonus* spp.

To test if *Arsenophonus* spp. can be transmitted vertically in honey bees, twenty additional queen-laid eggs were taken from each of three *Arsenophonus* spp. positive *A. mellifera* colonies, and subjected to two treatments as follows: (i) ten eggs were surface sterilized in 3% sodium hypochlorite for 1 min and rinsed three times in distilled water for 1 min (Vaughn 1971), (ii) the 10 remaining eggs were not treated prior to DNA extraction (= control). In addition, to test if there is a relation between the presence of *Arsenophonus* spp*.* in queen's bodies and eggs, ten non-surfaced-sterilized eggs from all eight *Arsenophonus*-positive colonies were individually analyzed. Extractions with Chelex^®^ solution from individual eggs, PCR and gel electrophoresis as well as sequencing, were performed under the same conditions as described above.

### Phylogenetic analyses

To get a first approach to the phylogenetic relationship of *Arsenophonus* spp. and the *Apis* hosts, the 16S rRNA sequences obtained from queens screening were aligned using the MUSCLE program (Edgar 2004a,b) and compared using the maximum likelihood method with the MEGA5.2 program (Tamura *et al*. 2011), under the Kimura two-parameter with a discrete gamma distribution model (K2 + G), because this model was the best suited one for our dataset by using the model testing option implemented in the MEGA5.2 program. The tree topology was evaluated by bootstrap resampling (1000 times).

## RESULTS

*Arsenophonus* spp. were the only endosymbiont tested positive in our samples consisting of eggs, workers and queens. It was detected in 24.2% of the surveyed *Apis mellifera* colonies (eight out of 33). This result is based on egg screening, in which *Arsenophonus* identity was confirmed by Sanger sequencing (see below). Since the worker screening was leading to false positive detections due to unspecific amplification of other gammaproteobacteria, e.g. *Gilliamella apicola*, it was not taken into consideration for the frequency analyses. All queens sampled from those *Arsenophonus*-positive colonies tested positive as well. *Arsenophonus* spp. were also detected in a single *A. dorsata* and *A. florea* queen (8.3%), but not in the 12 *A. cerana* queens.

The role that *A. mellifera* queens may play in the transmission of *Arsenophonus* spp. was investigated by analyzing (i) the location of *Arsenophonus* spp*.* in different queen body parts, (ii) the relation between the *Arsenophonus* spp*.* density in the queen's bodies and the number of *Arsenophonus* spp*.-*positive eggs and (iii) the location of bacteria within or on the surface of the eggs. First, the PCR-based diagnostics of the 16S rRNA gene sequences did neither detect *Arsenophonus* spp*.* in the ovaries of the queens (*N* = 8), nor in the spermathecae, thoraces and heads of surface-sterilized queens (*N* = 3). *Arsenophonus* spp*.* was only detected in the digestive tracts of the queens. Second, the qPCR-based assays (*yaeT* gene) indicate a poor relationship between the *Arsenophonus* spp*.-*positive eggs and respective loads in the queen's bodies (Pearson *r* = 0.066, *df* = 6, two tailed *P* = 0.88; Table S3, Supporting Informaiton). Third, in order to investigate whether *Arsenophonus* spp*.* could be transovarially transmitted, the presence of *Arsenophonus* spp*.* was PCR-diagnosed in surface-sterilized eggs (*N* = 30) and in untreated ones (*N* = 30), collected from three *A. mellifera* queens (Table S3, Supporting Informaiton). While 41% of untreated eggs were positive for *Arsenophonus* spp*.*, the bacteria were not detected in any of the surface-sterilized eggs (Pearson Chi Square test, *χ*^2^ = 15, *df* = 1, *P* < 0.001).

**Figure 1.**
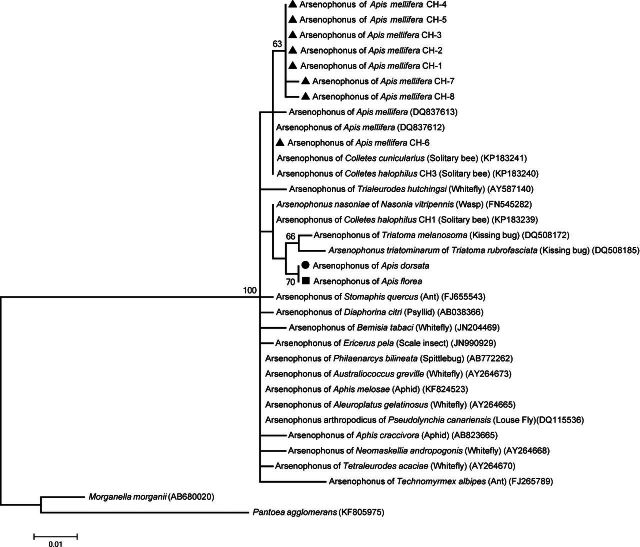
Maximum likelihood tree of *Arsenophonus* spp. isolates from *A. mellifera* (filled triangle), *A. dorsata* (filled circle) and *A. florea* (filled square) queens. The 587 bp 16S rRNA alignment from the sampled queens and isolates from other *Arsenophonus* were retrieved from the NCBI-GenBank. *Morganella morganii* and *Pantoea agglomerans* (Enterobacteriaceae) were used as outgroups. The bar indicates the genetic distance scale (number of nucleotide differences per site). Bootstrap values above 50 are shown in the corresponding nodes.

The amplicons originating from the 16S rRNA gene from the eight *A. mellifera* queens and from the single positive queens of *A. dorsata* and *A. florea* were sequenced. The *Arsenophonus* spp. identity was confirmed by high similarity (98%–99%) to sequences deposited in GenBank (accession numbers: , ). The phylogenetic tree shows that all sequences from the honey bee queens cluster together with previous sequences from honey bee workers and other insect hosts of *Arsenophonus* (Fig. 1). Interestingly, despite the low bootstrap support inside the *Arsenophonus* clade, the *Arsenophonus* spp. sequences originating from the Asian *A. dorsata* and *A. florea* are grouped together (bootstrap value 70%).

## DISCUSSION

The absence of *Wolbachia*, *Spiroplasma* and *Rickettsia* in our samples supports a low and/or seasonal prevalence of these bacteria in the genus *Apis*. *Arsenophonus* spp. was found in *A. mellifera* (24.2% of colonies) and also in queens of *A. dorsata* (8.3%) and *A. florea* (8.3%), but not in *A. cerana*. The phylogenetic analyses and low prevalence in sympatric *A. cerana* suggest that horizontal transmission from other honey bees is unlikely to be the source of *Arsenophonus* spp. in *A. dorsata* and *A. florea*. Instead, it appears as if *Arsenophonus* spp. are repeatedly acquired from the environment. The data also show that transovarial transmission of *Arsenophonus* spp. is unlikely in *A. mellifera*.

The low incidence of *Arsenophonus* and the non-detection of *Wolbachia*, *Spiroplasma* and *Rickettsia* in the four honey bee species confirm the sporadic presence of those bacteria in the genus *Apis*. The non-detection of *Wolbachia* and *Rickettsia* is in line with the most comprehensive microbial surveys in *A. mellifera* (Cox-Foster *et al*. 2007; Martinson *et al*. 2011). The only report of *Wolbachia* in European *A. mellifera* is based on PCR results, but unfortunately without confirmation by sequencing (Pattabhiramaiah *et al*. 2011). *Wolbachia* has been reported in African honey bees (Jeyaprakash, Hoy and Allsopp 2003; Jeyaprakash, Hoy and Allsopp 2009); nevertheless, its occurrence in European and Asian honey bees is still uncertain. The absence of *Spiroplasma* spp. in our tested colonies and queens may in part be explained by strong seasonal and regional variation of *Spiroplasma* species in honey bees as documented in Brazil and the USA (Schwarz *et al*. 2014).

The occurrence of *Arsenophonus* spp. in colonies/queens of *A. mellifera* (24.2%), *A. dorsata* (8.3%), *A. florea* (8.3%) and *A. cerana* (0 %) is in agreement with previous reports showing an irregular pattern of incidence of this bacterium among honey bee colonies (Babendreier *et al*. 2007; Cornman *et al*. 2012). In general, the incidence of the genus *Arsenophonus* in field collected insects is estimated to be around 5% (Duron *et al*. 2008), and its prevalence can also vary between populations of the same host species. For instance, in the yellow crazy ant (*Anoplolepis gracilipes*) the incidence of *Arsenophonus* spp*.* varies from 0%–50.8 % between different populations (Sebastien, Gruber and Lester 2012). Therefore, the differential prevalence levels of *Arsenophonus* spp*.* in the four studied *Apis* species are well within the previously reported variation.

The detection of *Arsenophonus* spp. in mated queens highlights a possible vertical transmission pathway mediated by the queen reproductive organs. However, the absence of *Arsenophonus* spp. from the ovaries, the spermathecae and sodium hypochlorite treated eggs, taken together with the non-significant correlation between the *Arsenophonus* spp. loads of queens and the number of positive eggs, do not support a transovarial transmission pathway that supposes the acquisition of the bacteria during oogenesis (Burgdorfer and Varma 1967). For secondary (facultative) endosymbionts, the colonization of the host's ovaries is frequent (Kose and Karr 1995; Goto, Anbutsu and Fukatsu 2006; Matsuura *et al*. 2012; Genty *et al*. 2014), but not exclusive, as secondary endosymbionts could also freely circulate in the hemolymph of the insect host (Cheng and Aksoy 1999; Goto, Anbutsu and Fukatsu 2006). Therefore, the detection of *Arsenophonus* spp. in the digestive tract of *A. mellifera* queens and on the surface of non-sterilized eggs suggests that, if vertically transmitted, the transmission might occur, for instance, during the oviposition (transovum) (Andreadis 1987). However, exclusive horizontal transmission may also be possible as in case of *A. nasoniae* (Werren, Skinner and Huger 1986). Indeed, the presence of *Arsenophonus* spp. on the egg surface can be also explained by the horizontal transmission through contaminated combs and/or contact with nurse bees. Then, one may consider *Arsenophonus* spp. as part of the honey bee gut microbiota, which seems to be exclusively horizontally transmitted through contact with nestmates after emergence (Martinson, Moy and Moran 2012; Powell *et al*. 2014). There is also evidence that this bacterium can cross the gut barrier and circulate in the honey bee hemolymph, as it was previously found associated with milky white hemolymph symptoms traditionally attributed to *A. mellifera* filamentous virus infections (Gauthier *et al*. 2015). The finding of *Arsenophonus* spp. in *V. destructor* also implies that this mite might play a role as a vector in the horizontal transmission of *Arsenophonus* spp. between honey bees at both individual and colony level (Hubert *et al*. 2015). Indeed, horizontal transmission might also involve other bee species. Phylogenetic analyses of the *Arsenophonus* spp. from honey bees cluster together with those isolated from solitary bees such as *Megachile rotundata* (McFrederick, Mueller and James 2014), *Colletes cunicularius* and C*. halophilus* (Gerth *et al*. 2015). The phylogenetic analysis of our *Arsenophonus* spp. isolates from *A. mellifera* (Fig. 1) also supports these previous results. The findings of the bacterium on the body surface of honey bees (Aizenberg-Gershtein, Izhaki and Halpern 2013), as well as in corbicular pollen (Corby-Harris, Maes and Anderson 2014) support the potential for transmission between bees when collecting nectar and pollen from shared flowers.

In this scenario, the occurrence of *Arsenophonus* spp. in the digestive tract of honey bees may constitute an oral-faecal route of transmission for this bacterium. Similarly, the oral-faecal transmission has been suggested for *Wolbachia* in the leaf-cutting ant *Acromyrmex echinatior* (Frost *et al*. 2014). Oral-faecal transmission has particular potential in the social insects because of the high population density and hygienic behavior in their colonies. Further, research should be undertaken to clarify the impact of *Arsenophonus* spp. infections on honey bee health.

Regarding the honeybee's *Arsenophonus* from *A. dorsata* and *A. florea*, the phylogenetic analysis from the 16S rRNA gene sequences shows some degree of divergence with *Arsenophonus* from *A. mellifera* (Fig. 1). This might be explained by the large distance between geographical origins of the samples. In general, there does not appear to be a relation between *Arsenophonus* diversification and the social habits of the host as bees, ants or aphids. However, since enterobacteria can carry several variable rRNA copies (Moran, McCutcheon and Nakabachi 2008; Sorfova, Skerikova and Hypsa 2008), those results should be confirmed with the use of additional phylogenetic markers.

In conclusion, this study shows for first time the presence of *Arsenophonus* spp*.* in queens, belonging to three different honey bee species. Taken together, the data do not support the vertical transmission of these bacteria through the queen, but the occurrence in the bees’ guts rather support a horizontal transmission following contact with nest mates or contaminated wax combs.

## Supplementary Material

Supplementary Data
