# Supplementary material for: Endosymbiotic bacteria in honey bees: Arsenophonus spp. are not transmitted transovarially
Source: FEMS Microbiol Lett. 2016 Jun 7;363(14):fnw147. doi: 10.1093/femsle/fnw147 (PMC4941583; doi:10.1093/femsle/fnw147)
Supplement: Supplementary Data [file fnw147_supplementary_data.zip › Supplementary_material.docx]

**Supplementary material**

1. Tables and figures

**Table S1.-** Diagnostic primers used in this study.

| Name | Primer name | Sequence | | Size | Reference |
| --- | --- | --- | --- | --- | --- |
| *Wolbachia* | Wsp-F | | TGGTCCAATAAGTGATGAAGAAACTAGCTA | ̴ 600 | Hoy *et al*., 2003 |
|  | Wsp-R | | AAAAATTAAACGCTACTCCAGCTTCTGCAC |  |  |
|  | Wsp-deg F | | GCRTTTGGYTAYAAAATGGA | ̴ 320 | Jeyaprakash *et al*., 2009 |
|  | Wsp-deg R | | GCACCAWAAGAACCRAARTA |  |  |
| *Spiroplasma* | SpixoF | | TTAGGGGCTCAACCCCTAACC | 810 | Duron *et al*., 2008 |
|  | SpixoR | | TCTGGCATTGCCAACTCTC |  |  |
|  | BS1-F | | AAGTCGAACGGGGTGCTT | 976 | Meeus *et al*., 2012 |
|  | BS1-R | | TGCACCACCTGTCTCAATGT |  |  |
| *Rikettsia* | Ricketb-F | | GCTCAGAACGAACGCTATC | 900 | Gottlieb *et al*., 2006 |
|  | Ricketb-R | | GAAGGAAAGCATCTCTGC |  |  |
|  | R1 | | GCTCTTGCAACTTCTATGTT | 434 | Von der Schulenburg *et al*., 2001 |
|  | R2 | | CATTGTTCGTCAGGTTGGCG |  |  |
| *Arsenophonus* | Ars16S-F | | GGGTTGTAAAGTACTTTCAGTCGT | 800 | Duron *et al*., 2008 |
|  | Ars16S-R2 | | GTAGCCCTRCTCGTAAGGGCC |  |  |
|  | yaeT-qF | | TCGAGCGCTATTTTCAACG | 90 | This study^α^ |
|  | yaeT-qR | | GTTGGCCGCTCTTTTACTTG |  |  |
| *A. mellifera*  *Lys-1* | Lys-1 F | | ACCCGATAATTCAACGACGA | 471 | Harpur and Zayed, 2013 |
|  | Lys-1 R | | CATTCGACCCTGGTTCATTT |  |  |
| *A. mellifera*  18S rRNA | AJ307465-955F | | TGTTTTCCCTGGCCGAAAG | 62 | Ward *et al*., 2007 |
|  | AJ307465-1016R | | CCCCAATCCCTAGCACGAA |  |  |

^α^Primers suitable for qPCR were designed from the outer membrane protein assembly factor (*yaeT*) gene, according to a nucleotide alignment of 17 *Arsenophonus* sequences retrieved from GenBank (GU226783, GU226784, GU226785, GU226786, GU226787, KF453465, GU226794, JX188416, JX188419, GU226795, GU226791, KF453458, GU226789, GU226790, GU226788, GU226793, JF743380). The alignment was performed using MEGA5.2 program (Tamura *et al*., 2011).

Forward and reverse primers were designed in the conserved areas using Primer3 program (http://biotools.umassmed.edu/bioapps/primer3_www.cgi).

**Table S2.-** Scheme of the dissections performed to the tested queens in this study. (**×)** shows the dissected body parts in the sampled queens**.**

|  | **Dissected body parts** | | | | | | |
| --- | --- | --- | --- | --- | --- | --- | --- |
| **Queens** | Head | Thorax | Digestive tract | Ovaries | Spermatheca | Body remains | Not dissected |
| *Apis mellifera* CH-1 |  |  | **×** | **×** |  | **×** |  |
| *Apis mellifera* CH-2 |  |  | **×** | **×** |  | **×** |  |
| *Apis mellifera* CH-3 |  |  | **×** | **×** |  | **×** |  |
| *Apis mellifera* CH-4 |  |  | **×** | **×** |  | **×** |  |
| *Apis mellifera* CH-5 |  |  | **×** | **×** |  | **×** |  |
| *Apis mellifera* CH-6 | **×** | **×** | **×** | **×** | **×** | **×** |  |
| *Apis mellifera* CH-7 | **×** | **×** | **×** | **×** | **×** | **×** |  |
| *Apis mellifera* CH-8 | **×** | **×** | **×** | **×** | **×** | **×** |  |
| *Apis cerana* (12 queens) |  |  |  |  |  |  | **×** |
| *Apis dorsata* (12 queens) |  |  |  |  |  |  | **×** |
| *Apis florea* (12 queens) |  |  |  |  |  |  | **×** |

**Table S3.-** *Arsenophonus* spp. quantification and occurrence in sterilized and non-sterilized eggs from *Arsenophonus*-positive *A. mellifera* queens.

| *Arsenophonus* positive queen | *Arsenophonus* DNA copies* / queen | Egg treatment | N | *Arsenophonus* positive eggs | *Arsenophonus* positive eggs [%] |
| --- | --- | --- | --- | --- | --- |
| CH1 | 6.47E+06 | Non- Sterilised | 10 | 3 | 30 |
| CH2 | 1.40E+07 | Non- Sterilised | 10 | 6 | 60 |
| CH3 | 5.29E+07 | Non- Sterilised | 10 | 4 | 40 |
| CH4 | 7.33E+06 | Non- Sterilised | 10 | 0 | 0 |
| CH5 | 5.45E+06 | Non- Sterilised | 9 | 5 | 55.6 |
| CH6 | 1.31E+07 | Non- Sterilised | 10 | 6 | 60 |
|  |  | Sterilised | 10 | 0 | 0 |
| CH7 | 9.03E+06 | Non- Sterilised | 9 | 5 | 55.6 |
|  |  | Sterilised | 10 | 0 | 0 |
| CH8 | 1.23E+07 | Non- Sterilised | 10 | 1 | 10 |
|  |  | Sterilised | 10 | 0 | 0 |

* *A. mellifera* 18S rRNA was used as an internal control to normalise for extraction efficiency.

The normalization was done according to Pfaffl (2001) and sample CH1 was used as calibrator.

2. References

Duron O, Bouchon D, Boutin S, *et al*. The diversity of reproductive parasites among arthropods: *Wolbachia* do not walk alone. *BMC Biol* 2008;**6**:27.

Gottlieb Y, Ghanim M, Chiel E, *et al*. Identification and localization of a *Rickettsia* sp. in *Bemisia tabaci* (Homoptera: Aleyrodidae). *Appl Environ Microbiol* 2006;**72**: 3646-3652.

Harpur B, Zayed A. Accelerated evolution of innate immunity proteins in social insects: adaptive evolution or relaxed constraint? *Mol Biol Evol* 2013;mst061.

Hoy M, Jeyaprakash A, Alvarez J, *et al*. *Wolbachia* is present in *Apis mellifera capensis*, *A. m. scutellata*, and their hybrid in Southern Africa. *Apidologie* 2003;**34**:53–60.

Jeyaprakash A, Hoy M, Allsopp M. Multiple *Wolbachia* strains in *Apis mellifera capensis* from South Africa. *Apidologie* 2009;**40**:178–183.

Meeus I, Vercruysse V, Smagghe G. Molecular detection of *Spiroplasma apis* and *Spiroplasma melliferum* in bees. *J Invertebr Pathol* 2012;**109**:172-74.

Pfaffl M. A new mathematical model for relative quantification in real-time RT-PCR. *Nucleic Acids Res* 2001;**29**: e45.

von der Schulenburg J, Habig M, Sloggett J, *et al*. Incidence of male-killing Rickettsia spp. (alpha-proteobacteria) in the ten-spot ladybird beetle *Adalia decempunctata* L. (Coleoptera: Coccinellidae). *Appl Environ Microbiol* 2001;**67**:270-277.

Ward L, Waite R, Boonham N, *et al*. First detection of Kashmir bee virus in the UK using real-time PCR. *Apidologie* 2007;**38**:181-190.
